# Perspectives of patients, first-degree relatives and rheumatologists on preventive treatments for rheumatoid arthritis: a qualitative analysis

**DOI:** 10.1186/s41927-018-0026-7

**Published:** 2018-07-05

**Authors:** Sarah Munro, Luke Spooner, Katherine Milbers, Marie Hudson, Cheryl Koehn, Mark Harrison

**Affiliations:** 1 Department of Family Practice, University of British Columbia, Vancouver, BC Canada; 2 Faculty of Pharmaceutical Sciences, University of British Columbia, Vancouver, BC Canada; 3 Centre for Health Evaluation and Outcome Sciences, St. Paul’s Hospital, Vancouver, BC Canada; 4 Division of Rheumatology, Jewish General Hospital and Lady Davis Institute, and Department of Medicine, McGill University, Montreal, QC Canada; 5 Arthritis Consumer Experts/JointHealth, Vancouver, BC Canada; 6 Arthritis Research Centre of Canada, Richmond, BC Canada

**Keywords:** Arthritis, Decision making, Discrete choice experiment, Focus groups, Health/economics, Rheumatology/economics, Rheumatoid/therapy

## Abstract

**Background:**

There is growing evidence that it may be possible to identify people at high risk of developing rheumatoid arthritis (RA). Assuming that effective interventions were available, this could mean that treatments introduced in the pre-symptomatic phase could prevent or delay the onset of the disease. Our study aimed to identify the potential attributes involved in decision-making around whether or not to take preventive treatment for RA, in order to inform the development of a discrete choice experiment (DCE) to ascertain consumer preferences for a preventive treatment program for RA.

**Methods:**

We conducted a focus group study to develop conceptual attributes, refine their meaning, and develop levels. Participants included RA patients, first-degree relatives of RA patients, and rheumatologists who were 18 years of age and over, could read and speak English, and could provide informed consent. Candidate attributes were refined through iterative rounds of data collection and analysis. All focus groups were audio-recorded and transcribed, and then analyzed using the Framework Method to identify, compare, and contrast key conceptual attributes.

**Results:**

Attributes identified from analysis included: *accuracy of the test, certainty in estimates, method of administration, risk of RA and risk of reduction with treatment, risk and seriousness of side effects, person recommending the test,* and *opinion of the health care professional.* Patients with RA, first-degree relatives of patients, and rheumatologists all valued the accuracy of testing due to concerns about false positives, and valued certainty in estimates of the test and preventive treatment. Patients and first-degree relatives desired this evidence from a range of sources, including discussions with people with the disease and health care professionals, and their preferences were modified by the strength of recommendation from their health care professional.

**Conclusions:**

The role of the person who *recommends a test* and the *opinion of a health care professional* are novel potential attributes involved in decisions around whether or not to take preventive treatment for RA, that have not been included in previous DCEs.

## Background

There is growing evidence that it may be possible to identify people at high risk of developing rheumatoid arthritis (RA) based on clinical and genetic risk factors, such as family history of RA [1], female sex [2–4], and lifestyle risk factors including smoking [5, 6]. Multiple studies have shown that RA has a prolonged and identifiable asymptomatic pre-clinical development phase, during which characteristic biomarkers appear, in particular antibodies against cyclic citrullinated peptide (CCP) [7–10]. These autoantibodies have been shown to precede the onset of RA symptoms by many years and are highly specific to RA [7, 11, 12]. The increasing evidence of a pre-clinical phase of RA has led to interest in the early identification of people at high risk of RA, and a possible window of opportunity for treatment in the pre-symptomatic phase to prevent or delay onset of the disease [13].

There is considerable uncertainty about when and whether those who are predicted to develop RA will actually develop RA [14], and if those individuals would be receptive to preventive treatment [15]. In this context, it is critical to explore the preferences of stakeholders who would be directly affected by preventive RA treatment in order to predict uptake of treatment options, and to inform policies for resource allocation [16, 17]. To the best of our knowledge, there are currently two qualitative studies involving first-degree relatives from the UK, Germany, and Austria [18, 19] and one exploratory binary choice experiment, which was not developed using qualitative methods [15], that explores or describes which attributes of a potential preventive treatment program for RA are valued by those who might be asked to recommend, consider, and provide or accept preventive treatment [17].

Discrete choice experiments (DCEs) are a rigorous method for exploring consumer preferences in health care services [18, 19]. In a DCE survey, the participant reviews a set of hypothetical scenarios and makes their preferred choice among two or more health care options. This technique allows the researcher to identify participants’ willingness to make trade-offs between the different characteristics, or “attributes,” of a health care service [19–24]. Undertaking qualitative work in participant populations before designing and administering a DCE is critical to identify important attributes and describe them in a way that is relevant and understandable, and to reduce the risk of inaccurate or biased DCE results [22].

Our study aimed to identify the potential attributes involved in decisions around whether or not to take preventive treatment for RA, to inform the development of a DCE that would subsequently be used to ascertain the preferences of people at risk of developing RA for development of a preventive treatment program for RA.

## Methods

### Study design

Following principles for qualitative attribute development in the design of DCEs [22], we conducted a focus group study to develop conceptual attributes, refine their meaning, and develop attribute levels.

### Setting and participants

Focus groups were conducted January to March 2016 in two large Canadian cities. Participants included RA patients, first-degree relatives of RA patients, and rheumatologists who were 18 years of age and over, could read and speak English, and could provide informed consent. Patient and first-degree relative participants were recruited through the marketing and communications lists of the Arthritis Consumer Experts/Joint Health group and the Arthritis Research Canada Arthritis Patient Advisory Board mailing list, whose subscribers are a general audience of arthritis patients. All first degree relatives were recruited via the mailing list or snowball sampling through the patient participants. Rheumatologists were recruited through personal invite from the rheumatologist on our team (MHu) and selected to represent a range of experience and geographic setting. Participants were screened for eligibility and provided with a consent and study information form in advance of the focus group. Ethical approval for the study was granted by the UBC Behavioural Research Ethics Board (H15–01948).

### Data collection

Iterative rounds of data collection and analysis were conducted to create the list of candidate attributes:

1. Round 1: One focus group each with RA patients, first-degree relatives, and rheumatologists to identify and refine the preliminary list of attributes, discuss clarity of description of RA, and consider potential treatment decisions around preventive treatment to be included in the DCE.
2. Analysis of focus group data and revision of the list of attributes based on participants’ feedback from the first round;
3. Round 2: One focus group each with patients and first-degree relatives (different participants for both groups to those in round 1) to provide feedback on the list of attributes and survey design. At the end of these focus groups participants were asked to rank the list of potentially important attributes as priorities.

Before commencing each focus group, the moderator reviewed the study information and consent form, and received written consent from each participant. A semi-structured interview guide was developed for the study consisting of questions and probes that explored potential candidate attributes, which were identified through consultation with a patient partner, rheumatologist and pharmacist experts, researchers, and a literature review. The review included existing choice-based studies of treatments for RA, either from the published literature or published as abstracts from the 3 previous years of American College of Rheumatology, European League Against Rheumatism, and British Society for Rheumatology meetings. *Conceptual* attributes – topics that may potentially, but not necessarily, be included in a DCE – that were identified prior to the first focus group included effectiveness at preventing RA [15], generalized and local side effects/adverse events [15, 23, 24], strength of evidence [24], how the treatments are taken [15, 23, 24], how often they are taken [15, 23, 24], how long they would be taken for [15], and cost [23]. These topics were explored through focus group discussion so that we could identify *candidate* attributes – concepts that are important, relevant, and understandable to people involved in decisions around whether or not to take preventive treatment for RA. Expert consultation consisted of discussion with clinical members of the research team in Vancouver regarding the types of information they would expect to communicate to a person making the decision.

Round 1 focus groups with first-degree relatives explored the potential impact of RA on their life, personal experiences of RA with relatives, their information needs, with whom they would discuss preventive treatment, and the importance of uncertainty in risk prediction, benefits, and incidence of side effects. In the second round of focus groups, first-degree relatives and patient groups reviewed and discussed a list of candidate attributes and asked to rank the importance of attributes with regard to the decision of whether to undertake preventive treatment and to eliminate attributes they felt to be unimportant. They also discussed the decision-making themes that arose from analysis of Round 1 focus groups and provided further feedback on the survey design. All focus groups were audio-recorded and transcribed for analysis. Finally, expert consultation consisted of discussion with clinical members of the research team in Vancouver regarding the types of information they would expect to communicate to a person making the decision.

### Data analysis

Qualitative analysis was guided by the Framework Method [25] to identify the key concepts emerging from each of the interview transcripts and to compare and contrast these across focus groups of patients, first-degree relatives, and rheumatologists. Analysis consisted of a) immersion in the transcripts, b) coding, c) development of a working analytical framework, d) applying the framework, e) charting the data into a matrix, and f) interpreting the data. Two researchers (LS, KM) read and re-read the transcripts to gain familiarity and then open-coded them independently line-by-line to identify salient themes. The two researchers then met to discuss their codes with a qualitative methods expert (SM), assess interrater reliability, make changes to labels for semantic consistency, and then grouped similar codes together to create a working analytical framework. This process of discussing the codes, testing them with the transcript data, and refining the framework was repeated until no new codes were generated. The coded transcripts were then entered into NVivo qualitative analysis software (version 10).

To complete the framework analysis, after data had been coded and organized, one researcher (LS) charted the data in a matrix table and identified illustrative quotes with a and b to indicate how well they represented the sub-theme. For each key theme, the matrix consisted of one row per participant group and one column per sub-theme. Potential attributes were generated from the matrices by interpreting them as a team (LS, KM, SM) and identifying patterns between participant groups and themes.

The final stage of analysis involved development of potential attributes and attribute-levels that would be important in considering whether to take or recommend preventive treatment. Through face-to-face discussion, the researchers (LS, KM, SM) engaged in data triangulation following constant comparison techniques [26]. We compared the framework analysis matrix with the potential conceptual attributes that had been identified through expert consultation and literature review. This triangulation sought to enhance the representativeness and legitimacy of attributes and levels developed. Throughout data collection and analysis, we engaged in strategies for quality and validity, including keeping an audit trail of memos, independent coding to reduce bias, and constant comparison of data with data.

## Results

### Summary of qualitative analysis and key themes

In total, 5 focus groups were conducted with 25 participants (13 patients [7 in first round, 6 in second round], 5 first degree relatives [3 in first round, 2 in second round], and 7 rheumatologists [second round]). The mean age among patients and first degree relatives was 50.4 (SD 18.3) and 29.4 (SD 12.4), respectively. Patients had experienced their disease on average 14.5 years (SD 9.1) and most (82%) were currently taking treatment for RA. The rheumatologists currently practiced in Alberta, British Columbia, Ontario, and Quebec. Analysis identified two key themes: “Living with Rheumatoid Arthritis” and “Preventing Rheumatoid Arthritis.” A summary of major themes and related subthemes is found in Table 1 and discussed below.

**Table 1:** Summary of Major Themes and Related Subthemes

Theme	Sub-Themes	
Living with Rheumatoid Arthritis	Living with RA	
	Being Proactive about My Health	
	Wanting a Better Quality of Life	
	Trying to Avoid the Side Effects of Medications	On Health (not deteriorating for patients)
		On Lifestyle (not getting worse for both patients and first-degree relatives)
	Having Concerns about the Impact of the Test	
Preventing Rheumatoid Arthritis	Questioning if Preventive Treatment Is Appropriate	
	Needing More Evidence	Due to uncertainty about the treatment
		Due to gaps in knowledge about RA
	Implementing Preventive Treatment for RA	In clinical practice with patients
		At the health system level
	Wanting Alternatives to Medication: For Preventive Treatment	

### Living with rheumatoid arthritis

For patients, *living with rheumatoid arthritis* involved coping with symptoms of joint swelling, without which one “might think [they] don’t have the disease.” The symptoms of the disease had an emotional impact on everyday life; for example, one patient felt “devastated” when they “couldn’t even for an afternoon get dressed, couldn’t pull up the zipper on [their] pants.” Positive emotional impacts included patients being *proactive about their health* and making lifestyle changes such as exercising or quitting smoking. In the second round of focus groups, patients further expressed that efforts to improve their quality of life also involved *trying to avoid the side effects of medication,* and some had strong perceptions that RA treatment had damaged their kidneys, liver, and heart. Patients had few *concerns about the impact of the test,* that is the potential impact of a positive result, for risk of RA and felt that any information gleaned from test results would enhance their health care experience. The results may also signal a wake-up call to change their relative’s health behavior and mitigate the future effects of living with RA, or seek further examination and testing before undertaking preventive treatment.

First-degree relatives’ perceptions of RA were very similar to that of patients and were shaped by their experience witnessing the effects of RA on their relative. One first-degree relative described RA as “not just a disease of the joints. It just starts in the joints. It can affect every organ in your body.” Some discussed *wanting a better quality of life* for their relative and described the lifestyle changes that happen when your family member lives with RA, such as no longer being able to travel. A minority of patients noted, however, that early information on their risk for RA would have negatively impacted their ability to enjoy their lifestyle while still asymptomatic. First-degree relatives indicated they would be interested in testing for RA, if it gave them concrete ways to *be proactive about their health* such as changing their lifestyle or taking preventive treatment to maintain their health. However, they had *concerns about the impact of the test*, particularly the potential damage of a false positive result on their mental wellbeing and their insurance coverage.

The rheumatologist focus group discussion suggested that one theme of primary importance to clinicians was *concerns about the impact of the test* for high-risk individuals. The lifestyle concerns raised by patients and first-degree relatives notably did not emerge amongst rheumatologists. However, they did share the practical concerns about the costs related to the test, as well as insurance and anxiety concerns stemming from a false positive result. Additionally, rheumatologists’ discussions highlighted the perceived challenge of supporting RA patients to *be proactive about their health*, particularly regarding medication adherence. One rheumatologist suggested that first degree relatives may be no more adherent to a preventive treatment regimen than their family members living with RA, questioning the utility of preventive testing and treatment. Rheumatologists agreed that maintaining current quality of life was an important factor in the consideration of treatment, though their preferences primarily involved avoiding side effects in the long term (Table 2).

**Table 2:** Thematic Framework for “Living with Rheumatoid Arthritis”

	Living with Rheumatoid Arthritis	Being Proactive about My Health	Wanting a Better Quality of Life	Trying to Avoid the Side Effects of Medication	Having Concerns about the Impact of the Test
Patient	“I was just devastated, I couldn’t even, for an afternoon get dressed, couldn’t pull up the zipper on my pants, and I just lost so much weight.”^b^	“You want to do something about it [RA]. It’s not just a matter of kind of thinking you’re going to get over it”^a^	“[Treatment would] minimize sort of like the long term impact of maybe like hunching and succumbing to the pain. So that was really good.”^b^	“And of course the medication is affecting all the other things, the liver, the kidneys. Your skin, your hair, like everything. Eyes.”^b^	“And you could have some kind of decision tree or flow chart. Okay, so I’ve got this positive marker, now what?”^b^
First Degree Relative	“She [family member] had a life and then once the disease came and took it from her, she didn’t [anything] anymore. She couldn’t do things.”^b^	“If there were perhaps a treatment that were extremely preventive and very effective at lessening the risk of developing such a disease, I absolutely would take the test because that to me leads to something that is preventive. That leaves me being able to take some action”^a^	“If that was a risk for the medication, it’s also a risk for the RA. You’re almost guaranteed to get serious infections and TB is completely likely. So would I rather get those now when I’m strong enough and healthy enough to fight them”	“Especially because of watching my mom with prednisone, if there’s anything that increase the mental risk, that would be like huge for me.”^a^	“And for me adding any kind of anxiety to it, not because [a test result] necessarily jars me into a realism that I’m not comfortable with, but because I don’t think it adds anything.”^b^
Rheumatologist		“They [first degree relatives] want to know [about RA risk] because they think that they can prevent disease in themselves.”			“Well, if I know I’m going to have Lupus then my insurance goes into the toilet, you know, and I don’t want that, so I don’t want to know. I don’t want my family to know.”^b^

^a^ and ^b^ next to quotes indicate moderate and high importance/representativeness, respectively

### Preventing rheumatoid arthritis

All participant groups *questioned if preventive treatment is appropriate* in first-degree relatives, due to the potential for serious side effects. Patients suggested to “start small” and “if the RA factor is rising, then look at something” rather than have a first-degree relative immediately take medication. Focus groups with first-degree relatives highlighted that if a trusted health care professional recommended the preventive treatment they would believe it was appropriate. All sought *more evidence* on the evidence for potential preventive treatments, including related to the dosing schedule, methods of administration, how it has been tested (i.e. clinical trials), short and long-term side effects of treatment, and the evidence on the effectiveness of the treatment in preventing RA. Patients were particularly hesitant about preventive treatment because they perceived there to be significant gaps in current knowledge and ability to diagnose and understand the causes of RA. Finally, when discussing how to *implement preventive treatment in practice*, analysis of all focus groups suggested cost was important and that counseling should emphasize the purpose and effectiveness of the treatment, to enhance adherence. At the time of diagnosis many patients were initially in denial of RA or believed that diet and lifestyle changes were needed, rather than medication.

First-degree relatives similarly focused on *wanting alternatives to medication*, such as natural “herbal” treatments. First-degree relatives also *needed more evidence due to uncertainty about the treatmen****t.*** This included topics such as the extent of which RA was hereditary, and the differences between RA and other types of arthritis, such as osteoarthritis. They perceived that the effectiveness and side effects of treatment for preventing RA might be similar to the treatment for their relatives living with RA.

The rheumatologist discussion focused on concerns about the appropriateness of preventive treatment due to the side effects of medications, and the *need for more evidence* on the probability that treatment would prevent RA. Analysis suggested rheumatologists have concerns about the marginal benefit of treatment compared to no treatment. Their discussions suggested that, in the absence of high quality data on the effectiveness of emerging pharmaceutical treatments, weight loss or smoking cessation would be reasonable treatments to recommend. When discussing how to *implement a preventive treatment program in practice*, the concerns of rheumatologists centered on the ability to identify people at high risk of RA, whether rheumatologists had capacity to take on more patients, and whether rheumatologists were well suited to provide preventive treatment interventions. Nevertheless, some rheumatologists were willing to consider providing preventive treatments in certain high-risk populations (i.e. first-degree relatives with RA, smokers, Aboriginal [First Nations, Inuit, and Métis] persons) (Table 3).

**Table 3:** Thematic Framework for “Preventing Rheumatoid Arthritis”

	Questioning if Preventive Treatment Is Appropriate	Needing More Evidence: Due to uncertainty about the treatment	Needing More Evidence: Due to gaps in knowledge about RA	Implementing Preventive Treatment for RA: In clinical practice with patients	Wanting Alternatives to Medication: For preventive treatment
Patients	“Because it [RA treatment] is going to stop your pain when you take it anyways, why would you want to take that before if it has a lot of risk involved?”	“How the treatment affects or it works, down to a cellular level. The methods and results of testing. All possible side effects, short-term, long-term, and complementary lifestyle choices.”^a^	“If we don’t know the cause [of RA], everything is suspect that we do. You know? And especially all the treatments”^a^	“People should know why they should take the drugs because, for people like me who were in denial or just thought I would eat better and exercise and do yoga and whatnot I’d be fine and I don’t need all these drugs.”	“Your whole generation just looks at so many different options.”^a^
First Degree Relatives	“From where it would be coming from, Dr.--- was like, ‘Hey, you know, there’s this treatment. You know, I know how badly it effects your mother. I think that you are possibly at risk for having it,’ and he suggested it to me, I would definitely take a look at it.”^b^	“There would always be that little bit in the back of my mind that would go, ‘Okay, how far is the treatment going to be advanced by the time that I get there.’ You know, like in another 15, 20 years of medical science how much is the treatment for people with it going to be advanced?”^b^	“And I’ve heard theories, everything from it [RA] skips generations to it’s immediate, to you know it only affects the women in one side of the family. I’ve heard a whole bunch of different crazy different things.”^b^		“So let’s say that it’s a 60% chance that it’s absolutely going to prevent rheumatoid arthritis later in my life, and there’s a herbal treatment which is, like, 55%, 50%. That massively changes what my personal treatment plan is.”^b^
Rheumatologists	“But from our point of view is it safe to say though that we, too, if there was good evidence that normalizing endosmosis, or that weight loss or smoking cessation reduces [RA], we would be more at ease with that sort of intervention than an intervention that involves medications with toxicity?”^b^	“I think that a really, really strong, good solid scientific placebo control or analyzed control, let’s do it, I’ll push for it. But before that it is do no harm and that is how I approach my patient.”^a^	“I think that if you’re able to profile rheumatoid as to those patients who have really terrible diseases, you know, you can get it under control … and you were able to give something really, I would feel that those patients that I would be willing to do [preventive treatment].”^a^	“Is there a marketing approach that would change actual behavior or compliance in all that sort of thing. I think that’s one thing medicine really hasn’t -- you know, drug companies do it all the time, but that is to sell drugs to us not to the patient”^b^	“Patients want a cure, and patients want a cure naturally, right? And natural is perceived as being with no risk, which is not always true.”

^a^ and ^b^ next to quotes indicate moderate and high importance/representativeness, respectively

### Discrete choice experiment attributes

Results of iterative focus group data collection and analysis suggested seven candidate attributes were involved in the decision to consider or recommend preventive treatment for RA: 1) accuracy of the test, 2) certainty in estimates of the risks and benefits of treatments and tests, 3) method of administration, 4) the initial baseline risk of RA and risk reduction with treatment, 5) risk and seriousness of side effects, 6) who recommends the person to consider treatment (e.g., a friend, or a health care professional), and 7) opinion of the health care professional about a preventive treatment option. Table 4 provides a summary of the final attributes and levels generated and modified through analysis of focus group data, as well as direct quotes from patients, first-degree relatives, and rheumatologists to support the selection of these attributes.

**Table 4:** Summary of potential attributes, their levels, and supporting quotes compiled from the Framework Analysis

Final Attributes	Lay Terminology	Key Quotations from Qualitative Data	Suggested Labels of Possible Levels
1) Accuracy of Test	How accurate is the test in predicting rheumatoid arthritis	“I guess I want to know how accurate the test is, and if there is any chance that you could maybe be told like oh, there is a very good chance of you getting it, but maybe finding out later that that actually wasn’t true.” – First-degree relative “Because if you don’t get IgA [rheumatoid factor], for example, up to 50%, first degree relatives would be positive and I doubt all those are going to get arthritis, so.” - Rheumatologist	• High • Medium • Low
2) Certainty in Estimates	How strong is the evidence for the test and preventive treatments	“Whether there was enough evidence to show that that treatment actually has a chance of preventing.” – Patient “Is there any data saying that coming from a high risk situation, what is the reduction?” – Rheumatologist	• Moderate • Limited • Very limited
3) Method of Administration	Whether it is an infusion, injection, tablet.	“You know, I went to Europe last year with my wife. We were gone for, you know, half a year. Now if I wasn’t able to do that because I had to go to a specific doctor twice a week to get this thing, no thanks. I’m good.” – First-degree relative	• Infusion • Injection • Tablet
4) Risk of RA and Risk Reduction with Treatment	The risk of developing rheumatoid arthritis without vs. with treatment	“Me personally, never [would consider testing]. Unless it’s 100% positive. Just with the test turn out.” - Patient “[I would consider testing] if there were perhaps a treatment that were extremely preventive and very effective at lessening the risk of developing such a disease.” – First-degree relative	• High • Medium • Low
5) Risk and Seriousness of Side Effect	The risk of a side effect from treatment	“And I’ve had side effects with - I had a heart attack. I had my kidneys at stage - just the stage before. I needed to have dialysis, so. You know, there is side effects that you get that you have to watch out for.” - Patient “Especially because of watching my mom with prednisone, if there’s anything that increase the mental risk that would be like huge for me.” - First-degree relative	• Major irreversible; minor reversible • Major reversible; minor reversible • Minor reversible
6) Who Recommends	Whether it is a health care professional, patient, or relative who recommends it	“[If I] learn that I had a high risk of developing RA, I would probably talk about it to people and then that is why I came up with who recommends it being important. And I think I would have to hear it from at least two sources to act on it,” – First-degree relative	• Health care professional • Patient • Relative
7) Opinion of Health Care Professional	Whether a health care professional or patient supports/wants to take test and/or preventive treatment	“I think that I also have a lot of trust at this point in what health care professionals say. And a lot of my own opinions, and ultimately in the end, like it would be my own opinion, but I just think a lot of my own opinion would come from what the doctor said” - First-degree relative	• Health care professional doesn’t prefer • Health care professional is neutral • Health care professional prefers

Patients and first-degree relatives valued the “Accuracy of the Test,” which they perceived to mean having a low rate of false positives. Rheumatologists were also concerned with the accuracy of the individual components of the test in predicting RA (i.e. genetics, presence of RA specific antibodies). Similarly, “Certainty of Risk Estimates” all groups agreed that the strength of evidence to support testing and preventive treatment was important. Rheumatologists particularly valued evidence from placebo-controlled trials in high-risk populations, and preventive treatments with the greatest risk reduction. These attributes were closely related to the themes of *Preventing Rheumatoid Arthritis* discussed above.

In considering how to operationalize the sub-theme *Wanting a Better Quality of Life* into an attribute for the DCE, our data suggested that potential candidates included risk and type of side effects, ability to maintain employment, and ability to maintain an active lifestyle. These attributes, however, had the potential to overlap with one another and not be mutually independent. Thus, in our second round of focus groups we were able to explore this theme further with participants to identify two key mutually exclusive attributes: “Method of Administration” and “Risk and Seriousness of Side Effect.” These attributes reflected our finding that all participant groups valued information on whether preventive treatments would be given as a tablet or injection, require regular monitoring from a health care professional and have side effects both in the short term and long term. They focused largely on wanting to know if treatments would limit their travel, employment, and sports/activities. All groups valued having information on the first-degree relative’s “Risk of RA and Risk Reduction with Treatment,” and rheumatologists in particular noted the value of information on the marginal benefit of treatment compared to no treatment.

Patients and first-degree relatives placed high value on “Who Recommends” the testing and treatment program, as well as “The Opinion of the Health Care Professional.” First-degree relatives wanted multiple perspectives from individuals who had experience with RA including patients, nurses, physicians, and rheumatologists. Data from our Round 2 focus groups with first-degree relatives reinforced this observation that the opinion of the health care professional was highly valued by first-degree relatives. These attributes were related to the sub-theme of *Needing More Evidence*, discussed above. The cost of testing and treatment was an attribute directly identified by both first-degree relatives and patients that we chose to exclude from our proposed DCE as there is significant variation in costs associated with medications in Canada depending on patient insurance coverage.

Finally, analysis of focus groups revealed how participants might interpret the attributes and levels, what lay terms are most understandable, their thought process in making trade-offs between different attributes, and personal experiences that may modify perceptions and values of RA. These decision-making processes and personal characteristics have been integrated into our survey development and analysis. For instance, demographic questions about age, sex, ethnicity, and, if surveying first-degree relatives, which of their relatives have RA have been included. We also include questions on attitudes to risk, preventive behaviours, and the importance of shared decision-making and discussion of cost in treatment decision-making. The background information also includes recorded information sections on RA, the attributes of the DCE and the process of completing the DCE survey, based on focus group discussions and feedback.

## Discussion

Our study suggests that patients with RA, first-degree relatives of patients, and rheumatologists may value and make trade-offs between seven different attributes when making the decision for testing and preventive treatment for RA. All valued the accuracy of testing, due to concerns about false positives, and valued certainty in estimates of the test and preventive treatment, due to perceptions that treatment and testing for preventing RA is based on emerging evidence. Patients and first-degree relatives desired this evidence from a range of sources and their preferences were modified by the strength of recommendation from their health care professional. The method of treatment administration was important for patients and first-degree relatives due to lifestyle concerns, while rheumatologists were concerned with how method influences adherence to treatment.

Previous DCEs involving RA decisions have been administered to the general population [24], those at high risk of developing RA [15], patients [23, 27, 28], and rheumatologists [29], with the majority focusing on treatment preferences [15, 23, 24, 27, 28]. One exploratory binary choice experiment sought the preferences of those at high risk of developing RA, finding that those individuals preferred treatments that had high reduction in the risk of developing RA and low risk of a serious side effect [15]. This study was informed by a qualitative exploration of the perspectives of first degree relatives in the context of willingness to enroll in preventive treatment trials, which was different from the population completing the choice experiment [32]. Nonetheless, their qualitative findings are similar to ours and suggest relatives prefer to avoid injections/impact on lifestyle and risks of side effects, experience uncertainty about whether they will get disease/if a treatment will help, and prefer prevention via routes other than drugs. However, the study did not explore the potential inaccuracies in testing for risk of RA. Furthermore, it did not seek to explore the tradeoffs made between treatment *attributes*, which may vary depending on the treatment available. A semi-structured interview study involving 34 first degree-relatives of RA patients in the UK, Germany, and Austria found, similar to our study, concerns about the certainty of predictive testing and expectations for need for support and opinions when considering and interpreting test findings [16]. However, this study did not investigate the preferences of RA patients and care providers. Thus, our research provides critical, novel evidence on the attributes of choice of preventive RA testing and treatment, not only from the perspective of those at risk of developing RA (first-degree relatives), but also from RA patients and rheumatologists who may be involved in the decision-making process. While our literature review of previous DCE’s included five of the seven candidate attributes identified through our study, our analysis further identified *who recommends the test,* and *the opinion of the health care professional* as potential attributes important to patients and their first-degree relatives. This reflected participants’ trust in their health care provider and increased willingness to try a test or preventive treatment that provider recommended.

The variation we observed between the values of rheumatologists and patients/first-degree relatives highlights the importance of eliciting patient preferences at the point of clinical decision-making and incorporating those preferences into care. Patient preferences for testing and treatment for preventive RA may be influenced by their firsthand experiences, the advice of important others, the strength of the recommendation from their care provider, and the quality of the evidence. A model of shared decision-making may prove useful for creating a mutual understanding between patients and providers of the factors that modify their preferences such as, for instance, the lifestyle factors that may influence adherence to treatment [30].

This study is strengthened by its systematic qualitative approach to the development attributes and attribute-levels for a DCE, a process that is recommended but typically either not conducted or is underreported in the literature [31–38]. A previous systematic review of 254 published DCEs in health care found that 44% (*n* = 111) did not mention at all any use of qualitative methods [38]. Our approach may be adapted for future DCE development studies. Further, including multiple perspectives in our focus groups may allow for the development of a generic DCE that can be administered with patients, first-degree relatives, and health care professionals and allow for direct comparison of preferences. Inclusion of multiple perspectives also mitigates the risk of excluding or missing important attributes, and avoids researcher bias which could occur if attributes were elicited from a literature review alone [22, 33].

One potential limitation, as with all qualitative studies, is that findings may not be adaptable to all jurisdictions, only to populations and health systems similar to our sampling frame. The first-degree relatives in this sample may be more highly engaged and educated because of familiarity with their relative’s disease, however the patient population was drawn from the general public of arthritis patients. We did not initiate a third round of focus groups to conduct a final review of the selected list of attributes and their levels, and instead relied on the involvement of our patient partner to ensure that the language we used was appropriate for a lay audience, evoked the conceptual labels identified in our qualitative analysis, and to confirm that the attributes were mutually exclusive with no conceptual overlap. The small size of the first-degree relative groups was due to drop out (two participants did not show and were lost to follow-up). To ensure saturation in spite of small sample size, probing and discussion in the first-degree relative groups continued until the researchers felt they had a full understanding of the participants’ perspectives and there were sufficient examples in each category to identify the characteristics of attributes [39]. We will also assess the comprehensibility and usability of the lay language descriptions when piloting our DCE survey in a small online sample.

Our focus group method offers a novel approach to attribute development and, in comparison to one-on-one interviews alone, offers the opportunity for participants to share different experiences and generate shared understanding. At the analysis stage, our Framework Analysis approach allows for synthesizing multiple perspectives and clear documentation of choices made by our research team about inclusion/exclusion of potential attributes and meaning of attributes. This meticulous process may be adopted by other research teams to enhance the rigour and transparency of choice experiment designs.

## Conclusion

This qualitative study involved exploring patient, first-degree relative, and rheumatologist preferences for preventive treatments and provides insight into the process of eliciting attributes through qualitative methods. Findings have shaped the development and administration of a DCE for choice of testing and treatment for RA, which will inform the development, design, and implementation of a preventive treatment programs for RA.

## Abbreviations

CCP: Cyclic Citrullinated Peptide
DCE: Discrete Choice Experiments
RA: Rheumatoid Arthritis
